# The association between life satisfaction, emotional support, and perceived health among women who experienced intimate Partner violence (IPV) – 2007 behavioral risk factor surveillance system

**DOI:** 10.1186/s12889-021-10665-4

**Published:** 2021-04-01

**Authors:** Vivian Hui, Rose Eva Constantino

**Affiliations:** Department of Health and Community Systems, School of Nursing, University of Pittsburgh, Pittsburgh, PA USA

**Keywords:** Life satisfaction, Emotional, Perceived health, Intimate partner violence

## Abstract

**Background:**

Intimate partner violence (IPV) is a pressing phenomenon whose consequences are associated with severe physical and mental health outcomes. Every minute, around 24 people in the United States are raped, physically injured, or emotionally abused by their intimate partner. Although having experienced IPV is not modifiable, emotional support is a protective factor to prevent victims from committing suicide. The psychological state of IPV victims is critical in post-traumatic events and this is evidenced in numerous qualitative interviews. Therefore, the objective of this study is to explore the association between IPV with emotional support, life satisfaction, and perceived health status in the United States.

**Methods:**

This study analyzed the data from the 2007 Behavioral Risk Factor Surveillance System. Univariate analyses, multivariable logistic regression analyses, and ordinal logistic regression analyses were used to estimate the adjusted odds ratios (AORs) and 95% confidence intervals (95% CIs) for factors associated with IPV. Analyses were conducted using SPSS version 25.

**Results:**

The analyses show that there is a strong association between IPV experience and emotional support (AOR:1.810; 95% CI = 1.626–2.015). Participants who had either physical violence or unwanted sex with an intimate partner in the past 12 months have 2.28 higher odds to receive less emotional support and 2.05 higher odds to perceive poor life satisfaction. Also, participants who reported experiencing IPV were associated with (AOR: 3.12; 95% CI =2.68–3.62) times the odds of having ≥6 days more mentally unhealthy days in a month. For perceived health outcomes, people who had been threatened with violence by a sex partner have 1.74 (95% CI =1.54–1.96) times the odds of having poor perceived general health status. IPV survivors have 3.12 (95% CI =2.68–3.62) times the odds of having ≥6 days more mentally unhealthy days in a month.

**Conclusions:**

People reported with any IPV experience are more likely to receive less emotional support, perceive dissatisfaction in life, and poor health outcomes. This study shows the need for policies centered on the development of interventions that focus on mental health for those who have experienced IPV.

## Background

Intimate partner violence (IPV), includes physical violence, sexual violence, unwanted sex, and psychological aggression by an intimate partner [1], which is a multidimensional phenomenon with consequences of severe social, emotional, and cognitive impairment [2]. Nearly 30% of the women have experienced IPV and reported a significant impact on their daily functioning [3]. Such experiences include physical, sexual, emotional abuse, and threats. These exposures had a graded relationship with serious consequences on the victim’s physical and mental health [4]. The prevalence of IPV and its sequelae have profound health consequences, economic burden, and public health significance.

IPV is attributed to an array of multidimensional health outcomes. Women are three times more likely to be exposed to domestic violence compared to men [5] and are most likely to suffer poor physical and mental health ranging from hypertension, diabetes [6], and higher rates of HIV/AIDS infection [7]. A growing literature suggests that IPV can impede psychosocial development and trigger behavioral and mental health problems. Traumatic experiences like IPV can also affect emotional and psychological development, increasing vulnerability to mental health problems such as sleep disturbances [8], major depression, anxiety, posttraumatic stress disorder (PTSD) [9–11], and suicide attempts [12]. Also, borderline, narcissistic, and antisocial personality disorders are common among IPV perpetrators [13]. Therefore, the health consequences of IPV are far-reaching and multifaceted.

The physiologic and psychosocial consequences of IPV are also notable. Greater exposure to IPV is associated with higher household out-of-pocket medical costs and a financial burden to society. According to the Centres for Disease Control (CDC), the estimated annual cost of IPV is over 8 billion arise from increasing health expenses, and decreasing productivity [14]. Moreover, the health care cost burden usually sustains for at least 3 years after the exposure to IPV until the victims recover from the trauma and resume their work [15]. Importantly, however, the economic burden of IPV is underestimated since the previous research mostly focused only on inpatient or referral data.

Research on IPV has thoroughly addressed its negative consequences. However, this line of inquiry can hardly display how adversity can also positively transform IPV survivors, and thus a comprehensive picture of recovery is absent [16]. Some studies reveal that some survivors can develop better adaptation, positive thinking, and emotional and social recovery despite experiencing traumatic situations [17, 18].

Evidence has shown that the availability of social and emotional support is essential to mental and behavioral health in women who have experienced IPV [19, 20]. Adequate actual- and perceived support can act as a buffer against the development of PTSD in trauma-exposed victims [21, 22]. The psychological state of IPV survivors is critical in post-traumatic events and this is evidenced in numerous qualitative interviews [23]. Yet, there is a paucity of representative studies to compare the perceived life satisfaction, emotional support, and health status among IPV in the current state of the science. In the absence of such knowledge, the development of effective intervention strategies and treatment protocol to address such deficiencies in IPV will likely remain problematic.

## Methods

2007 Behavioral Risk Factor Surveillance System (BRFSS) survey was analyzed to assess the association between IPV and the outcomes of perceived emotional support, life satisfaction, and health status among women aged 18 years and over in the United States [24]. BRFSS is a state-based, cross-sectional survey, collected via telephone. Though 13 years have passed since the data was collected, this survey in 2007 contains the most recent module on IPV that covered across states in the United States.

Participants were asked based on the questions from the core component, and some from the optional modules [24]. BRFSS selects individuals randomly by dialing household telephone numbers and interviews only one participant per household [24]. To evaluate the primary independent variable, for the current study, the optional Intimate Partner Violence Module was used. This optional module has been applied to the states of Hawaii, Virginia, and West Virginia, and therefore the analysis depicted only three states where the IPV module was included.

### Measures

Our data were obtained from the 2007 BRFSS self-reported survey, which is the latest BRFSS study that included an IPV module. IPV is measured by the following questions:

#### Intimate partner violence (IPV)

“Has an intimate partner ever threatened you with physical violence? This includes threatening to hit, slap, push, kick, or hurt you in any way.”

“Has an intimate partner ever attempted physical violence against you? This includes times when they tried to hit, slap, push, kick, or otherwise hurt you, but they were not able to.”

“Has an intimate partner ever hit, slapped, pushed, kicked, or hurt you in any way?”

“In the past 12 months, have you experienced any physical violence or had unwanted sex with an intimate partner?”

The first three questions do not have a specific timeframe for the IPV experience, while the last question about unwanted sex is constrained with any experiences that occurred in the past 12 months. The following variables were used to correlate the association with IPV. *Emotional support* is measured with the following question ‘How often do you get the social and emotional support you need?’ and participants responded with an ordinal level of measurement, ranging from “always”, “usually”, “sometimes”, “rarely”, “never”, “don’t know”, “not sure”, and “refused”. We collapsed the responses from five to three levels, using only “always”, “sometimes” and “rarely” for a better fit with our analysis.

*Life satisfaction* is measured with the following question ‘In general, how satisfied are you with your life?’ Participants responded from “very satisfied”, “satisfied”, “dissatisfied”, “very dissatisfied”, “don’t know”, “not sure”, and “refused”. We collapsed the responses from four to two levels, as “satisfied” and “dissatisfied” as a dichotomous variable.

*Perceived general health status* is measured with the following question ‘Would you say that in general, your health status is?’ The response options were “excellent”, “very good”, “good”, “fair”, and “poor”

*Perceived physical health, mental health, and poor health* are measured by the following questions with the response options “__number of days”, “none”, “don’t know/not sure”, or “refused”. The items measured in days were classified as “none”, “1–5 days” or “6 days or more” in a month.

‘Now thinking about your physical health, which includes physical illness and injury, for how many days during the past 30 days was your physical health not good?

‘Now thinking about your mental health, which includes stress, depression, and problems with emotions, for how many days during the past 30 days was your mental health not good?’

‘During the past 30 days, for about how many days did poor physical or mental health keep you from doing your usual activities, such as self-care, work, or recreation?’ Additional covariates considered were race, education, employment, income, and age.

### Analysis

The frequency and demographic variables were described by univariate analyses, while the association between perceived emotional support, life satisfaction, unhealthy days, sociodemographic factors, and IPV were conducted by bivariate analyses with chi-square tests for nominal data. Multivariable logistic regression analyses were applied to predict the adjusted odds ratios (ORs) and 95% confidence interval (CIs). Ordinal logistic regression analysis was initially used to examine the relationship of IPV variables to each of the ordinally scaled variables. Since the proportional odds assumption is not satisfied, the continuation ratio approach was used to obtain the ORs and CIs for each transition of the choices in the variable. Data analysis excluded those missing data or recorded as “don’t’ know/not sure” With the large sample size in the dataset, and the IPV module is not compulsory for participants, it is anticipated that the missing data are at random. We used hot deck imputation to address the missing variables, which calculated the average score on an identified outcome variable by matching the score of other variables in the sample (i.e., donor variables). We used participants’ gender, education level, and race as the donor variables. Hot deck imputation provides less bias compared to mean imputation and is deemed as a better overall solution than the listwise deletion [25]. Statistical significance is indicated by a 2-sided *p*-value of <.05. Race, education, and employment were used as covariates for all dependent variables, while income and age were added exclusively for perceived health outcomes including physical, mental, and poor health. Analyses were conducted using SPSS version 25.

## Results

Demographics showed a sample size of *n* = 19,102 with the mean age of 48.32 for those with IPV experience, while 53.87 is the mean age for those without any IPV experience. 3240 (16.9%) participants indicated an IPV experience in their lifetime. Women (*n* = 11,665; 61%) comprised the majority of the sample. The sample included white (*n* = 13,490; 78.6%) and non-white persons (*n* = 3681; 21.4%). Majority of the participants graduated from high school or had some high school education (*n* = 7650; 41.1%), followed by college or more (*n* = 6339; 33.2%), and college 1–3 years (*n* = 5089; 26.7%) as shown in Table 1.

**Table 1:** Descriptive statistics on demographics and IPV from states of Hawaii, Virginia, and West Virginia: 2007 BRFSS, United States

	Any Yes	IPV (Count/%) No	Total	X^2^ or T-test Statistics
Race				
White	2348 (13.7%)	11,142 (64.9%)	13,490 (78.6%)	
Non-White	478 (2.8%)	3203 (18.7%)	3681 (21.4%)	*P* < 0.001
Age (years)				
Mean +/− SD	48.32 **±** 14.468	53.87 **±** 16.825	52.93 **±** 16.58	*P <* 0.001
Marital status				*P <* 0.001
Married	1405 (7.4%)	9865 (51.7%)	12,170 (59.1%)	
Divorced/Widowed	1119 (5.9%)	3538 (18.6%)	4657 (24.4%)	
Single	707 (3.7%)	2429 (12.7%)	3136 (16.5%)	
Highest education attained				*P <* 0.001
High school grad or less	1207 (6.3%)	6443 (33.8%)	7650 (41.1%)	
College 1–3 years	1006 (5.3%)	4083 (21.4%)	5089 (26.7%)	
College or more	1024 (5.4%)	5315 (27.9%)	6339 (33.2%)	
Employment status				*P <* 0.001
Employed	1982 (10.4%)	9047 (47.4%)	11,029 (57.8%)	
Unemployed	823 (4.3%)	2596 (13.6%)	3419 (17.9%)	
Retired	428 (2.2%)	4193 (22%)	4621 (24.2%)	
Annual income				*P <* 0.001
Less than $35,000	1379 (7.9%)	5148 (29.7%)	6527 (37.6%)	
$35,000 - < $75,000	981 (5.7%)	5139 (29.6%)	6120 (35.3%)	
$75,000 or more	659 (3.8%)	4050 (23.3%)	4709 (27.1%)	

*SD* standard deviation

Logistic regression analysis (Table 2) shows a significantly higher odds ratio to explain the association between IPV experience, received emotional support, and perceived life satisfaction. Table 3 shows that persons who had been threatened with violence by their partner have 1.81 (95% CI = 1.626–2.015) times the odds of rarely receiving emotional support compared to persons who had not been threatened with violence by their partner. Simultaneously, people who had either physical violence or unwanted sex from a partner have 2.28 (95% CI =1.99–2.62) times the odds of rarely receiving emotional support compared to persons who have not. In terms of life satisfaction, persons who have been threatened with violence have 1.834 (95% CI =1.658–2.027) times the odds compared to persons who have not been threatened with violence. Moreover, persons who had experienced either physical violence or unwanted sex with their partner have 2.05 (95% CI =1.78–2.37) times the odds of more dissatisfaction in life compared to people who do not have unwanted sex with a partner.

**Table 2:** Logistic regression between IPV and emotional support, life satisfaction and perceived health from states of Hawaii, Virginia, and West Virginia: 2007 BRFSS, United States

	^Emotional Support		^Life Satisfaction	^General Health			**Physical Health		**Mental Health		**Poor Health	
	Sometimes or rarely	Rarely	Dissatisfied	Good, fair, poor	Fair, poor	poor	≥ 1 day(s)	≥ 6 days	≥ 1 day(s)	≥ 6 days	≥ 1 day(s)	≥ 6 days
IPV (threatened)	1.77 ^a^ CI:1.17–1.34^b^	1.81^a^ CI:1.63,2.02^b^	1.83 CI:1.66–2.02	1.31 CI:1.15–1.49	1.28 CI:1.16–1.41	1.74 CI:1.54–1.96	1.6 ^a^ CI:1.48–1.81 ^b^	1.94 CI:1.72–2.20	2.29 CI:2.07–2.53	2.53 CI:2.25–2.85	1.43 CI:1.26–1.62	1.62 CI:1.40–1.85
IPV (attempted)	1.72 CI:1.56–1.90	1.75 CI:1.57–1.96	1.78 CI:1.6–1.97	1.30 CI:1.13–1.48	1.30 CI:1.18–1.44	1.57 CI:1.34–1.78	1.5 CI:1.35–1.67	1.84 CI:1.62–2.10	2.16 CI:1.94–2.40	2.40 CI:2.13–2.71	1.38 CI:1.21–1.57	1.57 CI:1.35–1.83
IPV (ever violent)	1.74 CI:1.58–1.90	1.82 CI:1.63–2.01	1.82 CI:1.66–2.01	1.25 CI:1.12–1.42	1.28 CI:1.17–1.41	1.73 CI:1.54–1.95	1.60 CI:1.45–1.77	1.86 CI:1.66–2.10	2.27 CI:2.06–2.51	2.60 CI:2.32–2.92	1.41 CI:1.25–1.60	1.61 CI:1.39–1.86
IPV (unwanted sex)	2.00 CI:1.74–2.29	2.28 CI:1.99–2.62	2.05 CI:1.78–2.37	1.48 CI:1.23–1.79	1.64 CI:1.41–1.85	1.48 CI:1.33–1.64	1.932 CI:1.68–2.22	2.09 CI:1.78–2.45	3.01 CI:2.61–3.46	3.12 CI:2.68–3.62	1.44 CI:1.23–1.70	1.67 CI:1.38–2.01

*a = odds ratio (adjusted)

*b = CI 95%

^=adjusted covariates (race, education, employment)

** = adjusted covariates (race, education, employment, income, age)

**Table 3:** Descriptive statistics for IPV and study constructs from states of Hawaii, Virginia, and West Virginia: 2007 BRFSS, United States

	Any IPV (Count/%)		Total	X^2^ or T-test Statistics
	Yes	No		
Emotional support				*P* < 0.001
Always	1213 (6.4%)	8216 (43.4%)	9429 (49.8%)	
Sometimes	1070 (5.7%)	4426 (23.4%)	5496 (29%)	
Rarely	941 (5%)	3057 (16.2%)	3998 (21.2%)	
Life satisfaction				*P* < 0.001
Satisfied	1069 (5.6%)	7626 (40.1%)	8695 (45.7%)	
Dissatisfied	2154 (11.3%)	8186 (43%)	10,340 (54.3%)	
General Health				*P* < 0.001
Excellent	519 (2.7%)	2930 (15.4%)	3449 (18.1%)	
Good	1027 (5.4%)	5208 (27.4%)	6235 (32.8%)	
Fair	936 (4.9%)	5063 (26.6%)	5999 (31.5%)	
Poor	745 (3.9%)	2609 (13.7%)	3354 (17.6%)	
Physical Health				*P* < 0.001
None	1693 (9%)	10,409 (55.1%)	12,102 (64.1%)	
1–5 days	715 (3.8%)	2782 (14.7%)	3497 (18.5%)	
> 6 days	809 (4.3%)	2472 (13.1%)	3281 (17.4%)	
Mental Health				*P* < 0.001
None	1593 (8.4%)	11,611 (61.4%)	13,204 (69.9%)	
1–5 days	704 (3.7%)	2323 (12.3%)	3027 (16%)	
> 6 days	904 (4.8%)	1765 (9.8%)	2669 (14.1%)	
Poor Health				*P* < 0.001
None	1070 (11.2%)	4418 (46.2%)	5488 (57.3%)	
1–5 days	494 (5.2%)	1499 (15.7%)	1993 (20.8%)	
> 6 days	606 (6.3%)	1484 (15.5%)	2090 (21.8%)	

*SD* standard deviation

As for perceived health outcomes, persons who had been threatened with violence by their sex partner have a 1.74 (95% CI =1.54–1.96) lower perceived general health status compared with persons who have not been threatened by their sex partner.

On the number of unhealthy days in a month reported by participants, those who had been threatened with violence by their sex partner have a 2.29 (95% CI =2.07–2.53) more mentally unhealthy days in a month compared with persons who have not been threatened with violence by their sex partner. Contemporaneously, persons who had experienced either physical violence or unwanted sex with a partner have a 3.12 (95% CI =2.68–3.62) is having at least 72 unhealthy days in a year could negatively impact performance as a parent, employee, or student.

## Discussion

Having 6 or more “unhealthy days” in a month is a difficult predicament for anyone, whether a parent, worker or student. Having at least 72 unhealthy days in a year could ruin one’s hopes of being a competent parent, worker, or student. The purpose of this study was to understand the association between IPV experience and perceived emotional support, life satisfaction, and health status. First, we examined the relationships among the variables, and then we used a regression analysis to understand the influence of the covariates (i.e., race, education, employment, income, age) towards the dependent variables (i.e., emotional support, life satisfaction, and perceived health status). Finally, we explored the association between IPV and the outcome variables (i.e., dependent variables) with adjusted covariates.

Results indicate a strong association between participants’ IPV experience and emotional support, suggesting that persons with IPV experience received less emotional support. The most significant finding is that physical violence or unwanted sex with an intimate partner in the past 12 months has a 2.28 higher odds of rarely receiving emotional support and 2.05 higher odds of perceived life dissatisfaction. This finding is similar to the conclusions made by Coker et al. (2003) [25] in a structural equation model that higher emotional support affects the health outcomes of IPV survivors in the long run. However, our analysis from this state-wide national survey can capture more unreported IPV experiences from random selection, which demonstrates higher generalizability with larger sample size and representation in different states. Our study also elicited the evidence that emotional support and life satisfaction varies between the different degree of IPV experience. The more severe the IPV experience (i.e., unwanted sex over the past year), the lower the emotional support received, and life satisfaction perceived.

We also found that participants who reported IPV experience (e.g., threatened, attempted, over violent, or unwanted sex) reported more unhealthy days in a month, with mental health as the most significant of reasons for having “unhealthy days”. We believe that IPV experiences could negatively affect the mood of women, frequent exposure to IPV experience contributes to an unhealthy relationship with their intimate partner. With the accumulated threats and violent experience, women may hide their feelings and themselves from the external environment. Although initially surprising, these results appear somewhat consistent with the findings from the World Health Organization (WHO) multi-country sample in 2003 that found women who reported IPV at least once in their lifetime also reported significantly more emotional distress, suicidal thoughts (ORs 2.9 [CIs: 2.7–3.2]), and suicidal attempts (ORs 3.8 [CIs: 3.3–4.5]), compared to women who did not experience IPV [26].

With the widespread use of mobile phones and technology, we believed that the perceptions and forms of IPV can shift or evolve over some time (e.g., cyberbullying, sexting threat, online coercion, etc.) Given that our data is from the last decade, the measurement from using a survey might not be able to capture all kinds of IPV experience at one time. Recently, social media has become an indispensable platform to share feelings and seek help in life events, including IPV. Massive public awareness related to IPV has been aroused through Twitter via different hashtags (#metoo, #notokay, #maybehedoesnothurtyou) [27]. During the COVID-19 pandemic in 2020, the physical IPV is exacerbated 1.8 fold higher (*p* = 0.01) when compared to the past 3 years 2017–2019 [28]. As social media is the only platform accessible for survivors in the lockdown, we believe that social media would be a potential avenue for providing emotional support and improving life satisfaction. Telehealth is one of the ongoing trends after the pandemic, however, the abused women might not be able to talk with healthcare professionals virtually if they are still living with their intimate partner. Therefore, the nature of self-disclosure text and massive #hashtags campaign on social media would provide a better way out to express negative emotions and seek support among other IPV survivors. Future research should leverage the skills of text mining like sentiment analysis, topic modeling, and network analysis to examine the emotional or informational support received by IPV victims on social media.

### Implications

Like public health, social justice, and human rights issues, IPV experience is associated with less emotional support, poor life satisfaction, and poor mental health outcomes. IPV experience can linger in people’s minds after physical wounds have healed, casting a long shadow of other physical and psychological problems throughout the lifespan. Therefore, the process of growth and pain can be inextricably associated with trauma recovery [29]. To combat the effects of IPV on emotional support and life satisfaction, enhancing biobehavioral attributes of resilience could facilitate reappraisal and create a sense of purpose in the face of IPV, thereby improving mental health and post-traumatic growth. Resilience literature demonstrated some individuals can bounce back and develop healthy relationships after suffering from stressful events like IPV [17]. Since resilience can be replenished [30], we suggest that enhancing an IPV survivor’s sense of control, coping skills, social support, and providing multiple online resources could improve resilience and reduce the threatening and traumatic effects caused by IPV. Previous evidence has shown that resiliency programs [31] including social support [32], safety planning [33], and other technology-based interventions can mitigate the far-reaching consequences of IPV [34].

Policymakers should focus on how to turn the victims to become survivors by improving their resiliency. For example, social media would be an interactive and resourceful platform to find IPV support groups, financial support, or shelter availability information around their neighborhood. Policymakers should provide more funding for non-governmental organizations (NGOs) or violence prevention advocacy groups to integrate the online resources and create a hub for IPV survivors to navigate themselves easier. Also, as victim-blaming is one of the serious online harassment when IPV victims disclose their stories, policymakers should devise policies to combat the victim-blaming culture and encourage empathetic and supporting messages both on the internet and in a real-life situation. For example, using positive #hashtags to empower victims, highlighting the survivor stories, and creating an emotional support hub for abused women.

### Limitations

One of the limitations of this study is the lack of the use of a weighting formula that could have re-balanced the data to reflect the population more accurately. However, the large sample size of the data set represents greater generalizability of the BRFSS to the U.S. population. We also acknowledged that the 2015 National Intimate Partner and sexual violence survey may capture more recent data on IPV, however, the BRFSS 2007 also captured the physical, psychological, and sexual violence and included more mental health indicators (i.e., perceived life satisfaction and perceived mental health). These indicators are important for researchers to plan mental health intervention strategies. Another limitation is that each variable measured uses a single question only. For example, *emotional support* is measured with the following question ‘How often do you get the social and emotional support you need?’ and participants responded with an ordinal level of measurement, ranging from “always”, “usually”, “sometimes”, “rarely”, “never”, “don’t know”, “not sure”, and “refused”. Then, we collapsed the responses from five to three levels, using only “always”, “sometimes” and “rarely” for a better fit with our analysis. Further, some of the questions did not include a specific timeframe to recall the IPV experience.

We are concerned about missing data which may affect the interpretation of the results. The use of the dataset in this study is in BRFSS 2007, are discussed in the following way 1) the interpretation of results, 2) the analysis procedure, and 3) hot-deck imputation provides less bias compared to mean imputation and is deemed as a better overall solution than the listwise deletion [25]. Self-reported data and recall bias are other limitations of the study. The strength of the study is it provides information on the effects of emotional support, life satisfaction, and perceived health status on IPV in the U.S. which gives guidance to future intervention studies.

## Conclusions

In conclusion, the consequences of IPV are enormous. In this study, persons who reported having experienced IPV were more likely to receive less emotional support, more likely to perceive dissatisfaction in life, and have poor health outcomes. Mental health encompasses various aspects that may lead a person who survives IPV to develop poor mental health. These indicators of poor mental health include depression, PTSD, suicidal ideation, sleep disturbance, and self-esteem issues [35, 36]. The results of this study suggest the need for person-centered policies on the development of interventions that focus on mental health for people who have experienced IPV.

Precision health intervention programs [37] could be designed to provide individualized information, education, and prevention strategies on IPV that will match diverse IPV survivors of all races, socioeconomic statuses, gender, and age. Constantino and Crane (2005), found that social/emotional support intervention in women experiencing IPV is effective in improving perceived social/emotional support and decreasing healthcare utilization. The perception of the availability of social/emotional support is significant to survivors of IPV [20]. When their perceived availability of social/emotional support is low, survivors of IPV lose their ability to attenuate the unhealthy consequences of IPV [20]. As such, future research should concentrate on increasing emotional support, resilience, and life satisfaction from the online or offline intervention (e.g., social media and community support groups).

## Data Availability

The original dataset for the current study is available on the Center for Disease Control and Prevention website. The datasets used and analyzed during the current study are available from the corresponding author on reasonable request. The raw dataset can be obtained from https://www.cdc.gov/brfss/annual_data/annual_2007.htm.

## Abbreviations

BRFSS: Behavioral Risk Factor Surveillance System
HIV/AIDS: Human immunodeficiency virus infection and acquired immune deficiency syndrome
IPV: Intimate Partner Violence
PTSD: Post-traumatic Stress Disorder
